# Re-refinement of the spliceosomal U4 snRNP core-domain structure

**DOI:** 10.1107/S2059798315022111

**Published:** 2016-01-01

**Authors:** Jade Li, Adelaine K. Leung, Yasushi Kondo, Chris Oubridge, Kiyoshi Nagai

**Affiliations:** a Medical Research Council Laboratory of Molecular Biology, Francis Crick Avenue, Cambridge CB2 0QH, England; b Department of Veterinary Biomedical Sciences, Western College of Veterinary Medicine, University of Saskatchewan, 52 Campus Drive, Saskatoon, Saskatchewan S7N 5B4, Canada; c California Institute for Quantitative Biosciences, University of California, Berkeley, Berkeley, CA 94720-3220, USA

**Keywords:** pre-mRNA splicing, snRNA–protein complex, Sm-site recognition, improving model accuracy

## Abstract

The core domain of small nuclear ribonucleoprotein (snRNP), comprised of a ring of seven paralogous proteins bound around a single-stranded RNA sequence, functions as the assembly nucleus in the maturation of U1, U2, U4 and U5 spliceosomal snRNPs. The structure of the human U4 snRNP core domain was initially solved at 3.6 Å resolution by experimental phasing using data with tetartohedral twinning. Molecular replacement from this model followed by density modification using untwinned data recently led to a structure of the minimal U1 snRNP at 3.3 Å resolution. With the latter structure providing a search model for molecular replacement, the U4 core-domain structure has now been re-refined. The U4 Sm site-sequence AAUUUUU has been shown to bind to the seven Sm proteins SmF–SmE–SmG–SmD_3_–SmB–SmD_1_–SmD_2_ in an identical manner as the U1 Sm-site sequence AAUUUGU, except in SmD_1_ where the bound U replaces G. The progression from the initial to the re-refined structure exemplifies a tortuous route to accuracy: where well diffracting crystals of complex assemblies are initially unavailable, the early model errors are rectified by exploiting preliminary interpretations in further experiments involving homologous structures. New insights are obtained from the more accurate model.

## Abstract 2

The structure of the spliceosomal U4 snRNP core domain has been re-refined following molecular replacement using the minimal U1 snRNP as a search model. RNA-omit maps show that the U4 Sm-site nucleotides AAUUUUU are bound to the seven Sm proteins SmF–SmE–SmG–SmD_3_–SmB–SmD_1_–SmD_2_ in the same manner as the U1 Sm-site nucleotides AAUUUGU except at the U-to-G substitution in SmD_1_.

## 1. Introduction   

The removal of noncoding sequences (introns) from precursor messenger RNA is an essential step in eukaryotic gene expression. It is catalysed by a large RNA–protein complex called the spliceosome (Wahl *et al.*, 2009 ▸). The spliceosome is built up from five types of small nuclear ribonucleoprotein particles (U1, U2, U4, U5 and U6 snRNPs) and additional non-snRNP proteins in a defined order and hierarchy. Central to each snRNP is its eponymous snRNA molecule. In the U1, U2, U4 and U5 snRNPs, a common set of seven Sm proteins (SmB/SmB′, SmD_1_, SmD_2_, SmD_3_, SmE, SmF and SmG) bind as a ring around a single-stranded, semiconserved U-rich sequence PuAU_4–6_(G/U)Pu called the Sm site within the snRNA, forming the snRNP core domain (Yu *et al.*, 1999 ▸; Pettersson *et al.*, 1984 ▸; Bringmann & Lührmann, 1986 ▸). Core-domain formation is a prerequisite for import into the nucleus, where the snRNPs mature after recruiting particle-specific proteins. We initiated the crystallographic study of the U4 snRNP core domain in order to understand the recognition of the semiconserved Sm-site sequences by a common set of Sm proteins and the selection of snRNP-specific proteins by the assembled core domain.

The core domain is readily reconstituted from purified Sm proteins and specific nona-ribonucleotides, such as AAUUUUUGA containing the U4 Sm site or AAUUUG­UGG containing the U1 Sm site, either with or without the flanking RNA helices (Raker *et al.*, 1999 ▸). We have reconstituted the human U4 snRNP core domain using the seven Sm proteins in their natural sequences, except for deletion of the RG-rich C-terminal tails of SmD_1_ and SmD_3_, and a fragment of U4 snRNA that included the flanking helices to the Sm site, Stem II and Stem III, which were modified distal to the Sm site to promote crystal contacts. By capping Stem II with a GNRA tetraloop and incorporating the tetraloop receptor in Stem III (Leung *et al.*, 2010 ▸; Cate *et al.*, 1996 ▸), crystals were obtained in space group *P*3_1_, with unit-cell parameters *a* = 248.0, *c* = 251.9 Å, containing 12 copies of the U4 core domain in the asymmetric unit related by 222 rotational and threefold translational noncrystallographic symmetry (NCS). The crystals diffracted X-rays to 3.45 Å resolution along the *c* axis and 4 Å resolution perpendicular to it, but suffered from tetartohedral twinning (Roversi *et al.*, 2012 ▸). The presence of twinning was initially masked by the anisotropy and translational NCS, so that derivative data sets at low resolution showed apparent *P*6_1_22 symmetry. As described in Leung *et al.* (2011 ▸), the structure was solved using MAD phases at 5.5 Å resolution in a reduced cell of dimensions *a* = 142.1, *c* = 146.1 Å in *P*6_1_22, which in effect approximated all of the noncrystallographic symmetries as crystallographic. The initial model built in the reduced cell was expanded to the true cell in *P*6_1_22 symmetry with three copies in the asymmetric unit related by translational NCS and expanded again to *P*3_1_ with 12 copies related by translational and rotational NCS. The model was refined with twinning in *REFMAC*5 (Murshudov *et al.*, 2011 ▸) under 12-fold NCS restraints to an *R*_work_ of 27.7% and an *R*_free_ of 32.1% at 3.6 Å resolution before publication (Leung *et al.*, 2011 ▸).

The previously reported model of the U4 core domain (Leung *et al.*, 2011 ▸; PDB entry 4v5u) will be abbreviated as ‘U4prev’ in the remainder of this paper. It showed the seven Sm proteins, which share the Sm fold of an N-terminal helix (H1) followed by a highly bent β-sheet of five antiparallel strands, forming a closed ring by the hydrogen bonding of their β4 and β5 strands across the subunit interface. The ring is stabilized by a continuous hydrophobic belt of conserved inward-pointing side chains from all of the subunits. The manner of ring formation, also found in the crystal structures of archaeal Lsm (Sm-like) proteins (Collins *et al.*, 2001 ▸; Mura *et al.*, 2001 ▸; Törö *et al.*, 2001 ▸), and the cyclic protein order SmD_3_–SmB–SmD_1_–SmD_2_–SmF–SmE–SmG were both as proposed by Kambach *et al.* (1999 ▸). U4 snRNA threads through the central hole lined by loops L3, L5 and L2 of the Sm fold from the flat face to the tapered face of the ring. On the flat face, Stem II is strongly bent and lies over the SmD_3_–SmB–SmD_1_ sector with the 5′-terminus pointing towards the SmD_1_ edge of the ring. On the tapered face, Stem III is inclined towards SmD_3_–SmB with the 3′-terminus wedged between SmF and SmE, and in this disposition (Leung *et al.*, 2011 ▸) it would obstruct the binding of the U1 snRNP-specific protein U1-70K to the ring (Pomeranz Krummel *et al.*, 2009 ▸; Kondo *et al.*, 2015 ▸). Inside the hole, the middle seven nucleotides of the Sm site are each bound to one Sm protein by forming stacking and hydrogen-bonding interactions with conserved residues at equivalent positions in loops L3 and L5. While this model accounted for many biochemical observations, such as the cross-linking of the first and third U to SmG and SmB, respectively (Urlaub *et al.*, 2001 ▸), we also noted certain inconsistencies. For instance, G125 was modelled as bound to SmF, but after refinement its base was too distant from key residues in L3 of SmF to form stacking and hydrogen-bonding interactions (Leung *et al.*, 2011 ▸).

Formation of the snRNP core domain *in vivo* is mediated by assembly chaperones: the PRMTS and SMN complexes (Battle *et al.*, 2006 ▸). Crystal structures representing two assembly intermediates were reported subsequent to Leung *et al.* (2011 ▸). The structure of the 6S complex (Grimm *et al.*, 2013 ▸; PDB entry 4f7u) at 1.9 Å resolution shows SmD_1_–SmD_2_–SmF–SmE–SmG forming a horseshoe-shaped pentamer stabilized by the PRMTS component pICln binding across the gap, mimicking the SmD_3_–SmB dimer. The other structure at 2.5 Å resolution (Zhang *et al.*, 2011 ▸; PDB entry 3s6n) shows two SMN components bound to the SmD_1_–SmD_2_–SmF–SmE–SmG pentamer with the gap open, ready to engage the snRNA before ring closure by the addition of SmD_3_–SmB. The same kind of inter-Sm protein hydrogen-bonding interactions as described for the U4 core domain are observed in these assembly intermediates. Comparison of U4prev with these structures at higher resolution alerted us to sequence-register errors in our homology modelling of SmE from the SmD_3_–SmB and SmD_1_–SmD_2_ dimer structures (PDB entries 1d3b and 1b34; Kambach *et al.*, 1999 ▸). Hence, we reassessed U4prev as a preliminary interpretation and resolved to obtain a more accurate structure of the U4 core domain by re-refinement.

More recently, heteroheptameric ring structures from the Sm/Lsm family have been obtained by molecular replacement from U4prev or have been found to show similarities to it. These include two Lsm1–7 complexes (Sharif & Conti, 2013 ▸), Lsm2–8 in complex with the 3′ fragment of U6 snRNA (Zhou *et al.*, 2013 ▸) and the minimal U1 snRNP (Kondo *et al.*, 2015 ▸). Among these, the closest homologue is the minimal U1 snRNP, which contains the seven Sm proteins with a truncated U1 snRNA bound in the ring and two additional proteins specific to the U1 snRNP (Kondo *et al.*, 2015 ▸). The constructs for the minimal U1 snRNP crystals that diffracted X-rays to 3.3 Å resolution were designed on the basis of a 5.5 Å resolution model (Pomeranz Krummel *et al.*, 2009 ▸). The structure was determined by molecular replacement from a superposition of the protein part in all of the NCS copies in U4prev, and following density modification (Cowtan, 2010 ▸) the model was completed.

Crystal structures of all seven human Sm proteins are now available at 2.0 Å resolution or better (Kambach *et al.*, 1999 ▸; Grimm *et al.*, 2013 ▸) to provide interatomic distance restraints for refinement. New tools for model building and refinement at low resolution have also been developed (DeLaBarre & Brunger, 2006 ▸; Keating & Pyle, 2012 ▸; Murshudov *et al.*, 2011 ▸; Nicholls *et al.*, 2012 ▸; Brown *et al.*, 2015 ▸). Using these means, we have re-analysed the U4 core-domain data starting from molecular replacement using the homologous region of the minimal U1 snRNP (Kondo *et al.*, 2015 ▸; PDB entry 4pjo). Comparison of the re-refined structure of the U4 core domain with the minimal U1 snRNP offers a consistent structural explanation for the specificity of Sm-site recognition by the Sm rings in U1, U2 U4 and U5 snRNPs; it also shows how the distinctive geometries of snRNP-specific snRNA outside the core can prevent noncognate accessory proteins from binding to the core domain. We review the case history of structure determination and re-refinement, and comment on our route to accurate structural interpretations starting from problematic amplitude data and low-resolution initial phases.

## 2. Methods   

### 2.1. Characterization of the diffraction data   

Data processing has been described in Leung *et al.* (2011 ▸) and statistics are summarized in Table 1 ▸. The correlation of intensities between randomly partitioned half data sets fell to ∼0.5 at 3.6 Å resolution (Evans, 2006 ▸), so that data to this resolution were included in the refinement (Karplus & Diederichs, 2012 ▸). Before refinement, the amplitudes were corrected for anisotropy with truncation in the weak direction to where *F*/σ(*F*) ≥ 3 (http://services.mbi.ucla.edu/anisoscale/; Sawaya, 2014 ▸), which occurred at 4.0 Å resolution perpendicular to the *c* axis.

**Figure 1:**
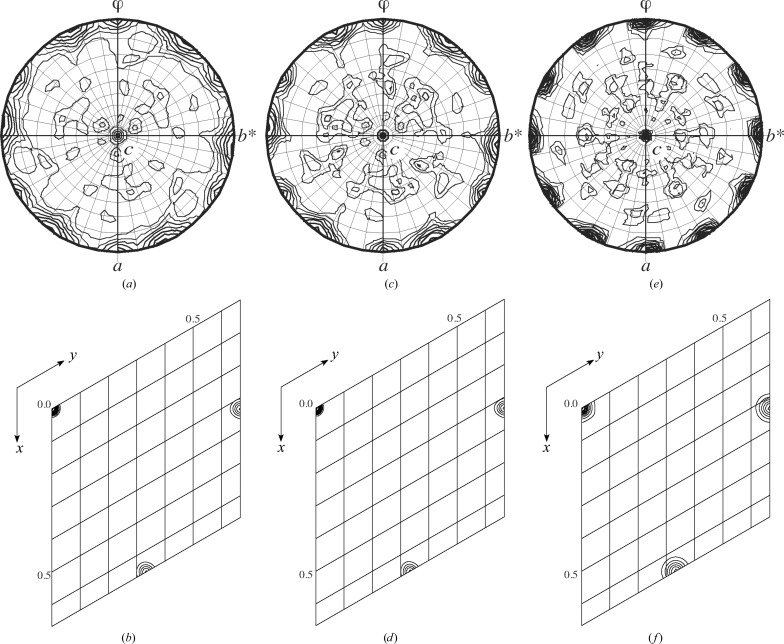
Noncrystallographic symmetries of the *P*3_1_ crystals. (*a*) κ = 180° section of the self-rotation map calculated in *MOLREP* (Vagin & Teplyakov, 2010 ▸) using a 30 Å integration radius and *F*_o_ to 6 Å maximum resolution. There are two sets of κ = 180° peaks in the *ab* plane of the trigonal crystallographic lattice, one set at φ = −37.1° with 61% of the origin peak height and the other at φ = −59.4° with 50% of the origin peak height, indicating the presence of twofold axes that are approximately mutually perpendicular, hence the approximate 222 rotational symmetry. (*b*) Native Patterson map calculated to 6 Å maximum resolution. The peak at (2/3, 1/3, 0) with 33.8% of the origin peak height indicates threefold translational NCS (translational pseudo-symmetry) in the [−1, 1, 0] direction. The rotational and translational NCS in combination generate 12 NCS copies per asymmetric unit. (*c*) and (*d*) were calculated as (*a*) and (*b*) but using *F*_o_ after anisotropy correction. The peak positions are unaffected by this correction, but the peak heights of self-rotation in (*c*) have decreased to 54 and 46% of the origin, respectively, and the peak height of the native Patterson in (*d*) has decreased to 32.4% of the origin. (*e*) and (*f*) were calculated after the re-refinement, using *F*_c_ that describe the untwinned model. In (*e*), the two sets of κ = 180° peaks in the *ab* plane, at φ = −28.8° with 81% of the origin peak height and φ = −58.11° with 79% of the origin peak height, accounted for the 222 rotational NCS detected in (*a*). In (*f*), the native Patterson peak at (2/3, 1/3, 0) with 46.4% of the origin peak height accounted for the threefold translational NCS detected in (*b*). The peaks in (*a*)–(*d*) are broadened by twinning.

Fig. 1 ▸ illustrates the noncrystallographic symmetries (NCS). The self-rotation map at 6 Å resolution showed κ = 180° peaks indicating 222 noncrystallographic rotational symmetry (Fig. 1 ▸ *a*). The native Patterson map at 6 Å resolution showed a peak at (2/3, 1/3, 0) indicating threefold translational NCS (translational pseudo-symmetry) in the [−1, 1, 0] direction (Fig. 1 ▸ *b*). In combination, they give rise to 12 copies of the U4 core-domain complex in the asymmetric unit, as reported in Leung *et al.* (2011 ▸). Correcting the anisotropy in *F*_o_ before refinement did not alter the NCS relationships (Figs. 1 ▸ *c* and 1 ▸ *d*), except that it slightly accentuated the deviations from approximate symmetries with increasing resolution. The presence of tetartohedral twinning caused peak broadening in the observed self-rotation and native Patterson maps compared with the same plots calculated using untwinned structure factors (Figs. 1*e* ▸ and 1 ▸ *f*).

### 2.2. Molecular replacement   

Two search models were constructed. (i) The ‘U1-derived’ model: from the minimal U1 snRNP coordinates (PDB entry 4pjo) containing four NCS copies, the copy having the lowest average *B* factors was selected, the U1-70K and U1-C proteins were deleted, the U1 snRNA was truncated to the nonamer AAUUUGUGG and each Sm protein was truncated to the Sm fold consisting of the N-terminal helix and a five-stranded β-sheet. (ii) The ‘U4–U1 hybrid’ model: the NCS copy with the lowest average *B* factors from U4prev and that from the minimal U1 snRNP were aligned by secondary-structure matching (Krissinel & Henrick, 2004 ▸) within the Sm fold, the SmF–SmE–SmG sector of U4prev was replaced as a block by its counterpart from the minimal U1 snRNP and the U4 snRNA and Sm proteins were truncated to positions equivalent to those in the U1-derived model. These two models differ crucially in the sequence and conformation of their RNA nonamer, corresponding to the Sm site. We carried out molecular replacement and preliminary refinement (§2.3) from both models in parallel until the U4 Sm site could be rebuilt.

Molecular replacement was carried out using *Phaser* (McCoy *et al.*, 2007 ▸) in the resolution range 66–5 Å. The U1-derived model found a solution set of 12 copies with an LLG (log-likelihood gain) of 9246; the U4–U1 hybrid model led to a similar solution set with an LLG of 5884. Both solution sets can be brought into a one-to-one correspondence with the 12 copies contained in U4prev by an origin shift along the *c* axis, indicating that the NCS relationships detected by molecular replacement are similar to those contained in the U4prev model.

### 2.3. Rigid-body and restrained refinement without the flanking RNA stems   

Phases from the molecular-replacement solutions were improved by rigid-body and restrained refinement in the presence of twinning using *REFMAC*5 (Murshudov *et al.*, 2011 ▸) prior to the calculation of OMIT maps (§[Sec sec2.4]2.4) with the objective of validating or remodelling the Sm site. The refinement was run until *R*_free_ ceased to decrease. During restrained refinement, automatically generated 12-fold local NCS restraints (local NCSR) were imposed. Secondary-structure restraints were not used. Automatic weighting between the X-ray and geometry terms was used except as otherwise stated. Electron-density maps were calculated with regularized sharpening (Nicholls *et al.*, 2012 ▸) in *REFMAC*5 to facilitate model building using *Coot* (Emsley *et al.*, 2010 ▸).

The molecular-replacement solution from the U1-derived model was subjected to rigid-body refinement at 5 Å resolution with one core-domain complex per rigid group for 60 cycles (*R*_work_ and *R*_free_ = 29.4 and 30.9%, respectively), followed by restrained refinement at 3.6 Å resolution with a matrix weight of 0.01 for 60 cycles (*R*_work_ and *R*_free_ = 24.0 and 27.0%, respectively). Similarly, the molecular-replacement solution from the U4–U1 hybrid model was subjected to rigid-body refinement at 5 Å resolution with one core-domain complex per rigid group for 40 cycles (*R*_work_ and *R*_free_ = 34.1 and 33.8%, respectively) and then one Sm protein or snRNA chain per rigid group for 20 cycles (*R*_work_ and *R*_free_ = 31.8 and 32.1%, respectively), followed by restrained refinement with a matrix weight of 0.01 for 20 cycles (*R*_work_ and *R*_free_ = 26.7 and 30.3%, respectively). The refinement established that there are four twin domains with somewhat unequal twin fraction (Table 1 ▸). Both models were rebuilt to remove Ramachandran and rotamer outliers.

### 2.4. RNA-omit maps and rebuilding of the Sm-site nona-nucleotide   

**Figure 2:**
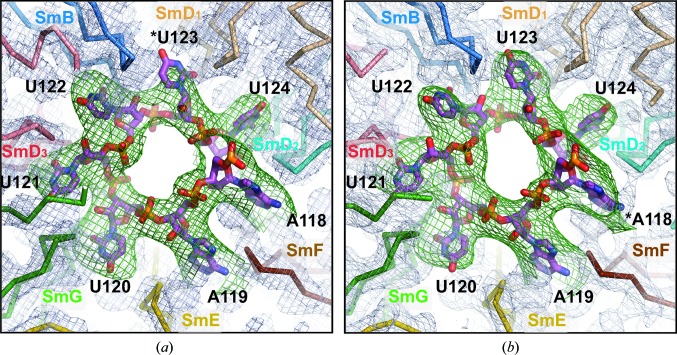
NCS-averaged OMIT maps of the Sm site superposed on the final coordinates of the Sm site. The OMIT maps (green) are calculated (§[Sec sec2.4]2.4) following molecular replacement, rigid-body refinement and restrained refinement by repeating the restrained refinement with the occupancies of the Sm-site nonanucleotide set to 0.01 and averaging the *mF*_o_ − *DF*_c_ density over the 12 NCS copies. (*a*) The U1-derived OMIT map contoured at 8.0σ (0.09 e Å^−3^) shows that at the G→U substitution U123 (marked by *) of U4 snRNA is bound in SmD_1_. (*b*) The U4–U1 hybrid-derived OMIT map contoured at 7.5σ (0.08 e Å^−3^) shows that the first A of the U4 Sm-site nonamer, A118 (marked by *), is bound in SmF. Figs. 2–5 and 7–10 were prepared using *PyMOL* (v.1.7.2; Schrödinger).

It was necessary to rebuild the snRNA in the Sm site given by the molecular-replacement models because the U1-derived model contained the U1 Sm-site nonamer with two sequence differences from the U4 Sm nonamer, while the U4–U1 hybrid model contained the Sm-site nonamer of U4prev, where we (Leung *et al.*, 2011 ▸) had detected an inconsistency at G125 (see §[Sec sec1]1). Therefore, we calculated two OMIT maps for the Sm site in both models by omitting the RNA nonamer from the output of the preliminary refinement described in §[Sec sec2.3]2.3 and carrying out a further 20 cycles of restrained refinement using the same protocol as in §[Sec sec2.3]2.3. Fig. 2 ▸ shows the pair of OMIT maps, which have been 12-fold NCS-averaged to enhance their signal-to-noise ratios.

The OMIT map originating from the U1-derived model (Fig. 2 ▸ *a*) generally agreed with that model except at the sixth position, where it showed little density over the base of G in U1 but showed good density near loops L3 and L5 of SmD_1_. It indicates that unlike the G of U1 snRNA, which lies outside the central hole (Kondo *et al.*, 2015 ▸), U123 at the sixth position of the U4 nonamer is bound inside the pocket in SmD_1_ as reported by Leung *et al.* (2011 ▸). The OMIT map originating from the U4–U1 hybrid model (Fig. 2 ▸ *b*) showed good density where the first A of the U1 nonamer would be stacked with Tyr39 of SmF. It implies that the equivalent A118 at the first position of the U4 nonamer is similarly bound in SmF, which contradicted our earlier interpretation that assigned G125 to SmF (Leung *et al.*, 2011 ▸). Therefore, the seven nucleotides of the U4 Sm site that are respectively bound by the Sm proteins SmF–SmE–SmG–SmD_3_–SmB–SmD_1_–SmD_2_ range from A118 to U124.

The Sm site of the U4 snRNA was rebuilt in the U1-derived model according to its OMIT map and refinement was continued from this model only because this model refined to lower *R* factors in the preliminary refinement (§[Sec sec2.3]2.3) compared with the alternative model and it required less rebuilding. We mutated the sixth and ninth nucleotides of the U1 nonamer to the U4 sequence and rebuilt U123 in the SmD_1_ pocket as reported by Leung *et al.* (2011 ▸). The refinement after rebuilding the Sm site decreased *R*_work_ and *R*_free_ to 24.1 and 26.6%, respectively. An elongated positive difference density emerged at the 3′-end of the nona-nucleotide, indicating the orientation of Stem III.

### 2.5. Modelling the flanking RNA stems   

In U4prev the 12 complexes are paired by the contacts between their flanking stem loops: when the tetraloop motif in Stem II of one complex contacts the tetraloop receptor motif in Stem III of another, Stem II of the latter contacts Stem III of a symmetry mate of the former. Such pairing also exists among the 12 complexes of the current model since they show a one-to-one correspondence with U4prev (§[Sec sec2.2]2.2). As a starting model, we copied the stem loops from one contacting pair in U4prev to all of the contacting pairs in the current model by secondary-structure matching over the corresponding Sm protein rings (Supplementary Fig. S1). Stem III of each complex was repositioned by rigid-body fitting of its 5′ strand within the proximal segment (before the inserted tetraloop-receptor motif) to the elongated positive difference density that extended from the 3′-end of the nonamer. The distal segment of the repositioned Stem III overlapped with loop L4 of SmB, and the latter was deleted. Stem II could not be positioned in the weak density to form the tetraloop-to-receptor interaction with Stem III (see Fig. 2*d* in Leung *et al.*, 2010 ▸). Instead, the tetraloop-to-receptor distances observed in the crystal structure of the group II intron (Zhang & Doudna, 2002 ▸; PDB entry 1kxk) or the group I ribozyme domain (Cate *et al.*, 1996 ▸; PDB entry 1gid) were used as external restraints (see below) in the first three rounds of refinement to favour the expected intermolecular contacts between Stem II and Stem III.

External restraints for interatomic distances within the Sm proteins were generated automatically using *ProSMART* (Nicholls *et al.*, 2012 ▸), with PDB entry 1d3b as a reference for SmD_3_ and SmB (Kambach *et al.*, 1999 ▸) and PDB entry 3s6n (Zhang *et al.*, 2011 ▸) or 4f7u (Grimm *et al.*, 2013 ▸) as a reference for SmD_1_, SmD_2_, SmE, SmF and SmG. When multiple reference chains were available for a target chain, the reference chain giving the best alignment score was selected for the calculation of restraints. The long L4 loops of SmD_2_ and SmB were excluded from the application of distance restraints because in the presence of RNA their conformations are likely to differ from the RNA-free reference structures. The application of external restraints on the protein part over the large number of cycles, which were required to remodel the RNA stems, reduced the overfitting (|*R*_work_ − *R*_free_| smaller by ∼1%) and improved the Ramachandran statistics (96% in favoured regions compared with 93%).

After three rounds of restrained refinement, each lasting 200 cycles, under these external restraints and ‘jelly-body’ restraints with a σ of 0.02 Å, alternated with substantial rebuilding of the RNA stems, these stems have shifted sufficiently so that all pairs of tetraloops and receptors made contacts, and the stems were joined to the single-stranded nonamer without stereochemical violations. Then, in accordance with the 2*F*_o_ − *F*_c_ map, the nucleotide in the 3′-most position in the nonamer, A126, was rebuilt into the *anti* conformation from the *syn* conformation of the equivalent G in the U1 nonamer. Base pairing was adjusted manually. Two more rounds of refinement, each lasting 100 cycles, followed during which the base-pairing and parallelization restraints automatically generated by the program *LIBG* (Brown *et al.*, 2015 ▸) were introduced but the tetraloop-to-receptor distance restraints and *ProSMART*-generated protein distance restraints were no longer used. Including the RNA stems in refinement decreased *R*_work_ and *R*_free_ to 17.8 and 22.7%, respectively.

### 2.6. Rebuilding loop L4 and extensions from the Sm fold   

Weak positive difference density alongside the RNA Stem III traced out the L4 (β3–β4) loop of SmD_2_, which was built and real-space refined in a 4*F*_o_ − 3*F*_c_ map. L4 of SmD_2_ consists of a β-ribbon up to residues Pro78 and Pro89 and a terminal loop with divergent conformations. L4 of SmB from the U1-derived model was deleted for Stem III to be built in its space, and there was density remaining for this loop to be rebuilt in only one copy.

The C-terminus of SmD_2_ is at the interface with β4 of SmF from an adjacent copy, and both elements were rebuilt to relieve clashes. At the N-terminus of helix H1 in SmD_2_ an additional helix (H0) was built into the difference density that points to a bend in Stem II between the first three G–C pairs and the G–U wobble pair. In SmD_3_, SmB, SmD_1_, SmE and SmF small extensions were built from their C-termini.

### 2.7. RNA backbone conformation   

Errors in the RNA backbone conformation detected by *MolProbity* (Chen *et al.*, 2010 ▸) were corrected by rotamerization of the existing structure using *RCrane* (Keating & Pyle, 2012 ▸) as implemented in *Coot*. However, the correction of the backbone torsion errors by *RCrane* was found to introduce bond-angle errors, while subsequent correction of bond-angle errors through regularization in *Coot* or refinement in *REFMAC*5 was found to reintroduce some of the backbone errors. We therefore generated external restraints for refinement from idealized RNA geometry as follows. The RNA part of the model after rotamerization by *RCrane* was copied out and subjected to 200 steps of conjugate-gradient minimization without experimental data using the model_minimization.inp task in *CNS* (Brünger *et al.*, 1998 ▸). During the minimization, dihedral angle values for the ribose ring in both 3′-*endo* and 2′-*endo* conformations were sampled and a harmonic restraint of 10 kcal mol^−1^ was applied on all atoms. The *CNS*-minimized (idealized) RNA coordinates have reduced bond-length and bond-angle errors while retaining the *RCrane*-corrected backbone conformation, and they were utilized as an external reference in *ProSMART* to generate self-restraints for the RNA part of the model. These self-restraints for RNA were applied in *REFMAC*5, in addition to the *LIBG*-generated base-pairing and parallelization restraints (with the weight scale of σ for the parallelization term set to 1.0), the jelly-body restraints and local NCS restraints, to refine the complete model of protein and RNA. After 20 cycles of *REFMAC*5, the regions of the 5′-stem that showed poor fit to the 2*F*_o_ − *F*_c_ map were rebuilt using *RCrane*. The cycle, from model minimization for the RNA part in *CNS* (using a reduced harmonic restraint of 5 kcal mol^−1^) and RNA self-restraint generation using *ProSMART* to 20 cycles of *REFMAC*5 under the combination of restraints, was repeated once. The final model showed an *R*_work_ and *R*_free_ of 17.7 and 22.4%, respectively, against data in the resolution range 66.1–3.6 Å (Table 1 ▸). *MolProbity* reported an all-atom clashscore in the 97th percentile and a *MolProbity* score in the 100th percentile compared with structures of similar resolution, and 97.7% of the RNA suites are in consensus backbone conformations (Richardson *et al.*, 2008 ▸; Keating & Pyle, 2012 ▸). The atomic coordinates of the re-refined U4 core domain have been deposited in the Protein Data Bank (PDB entry 4wzj).

## 3. Results   

### 3.1. Validity of the re-refined U4 core-domain structure   

**Figure 3:**
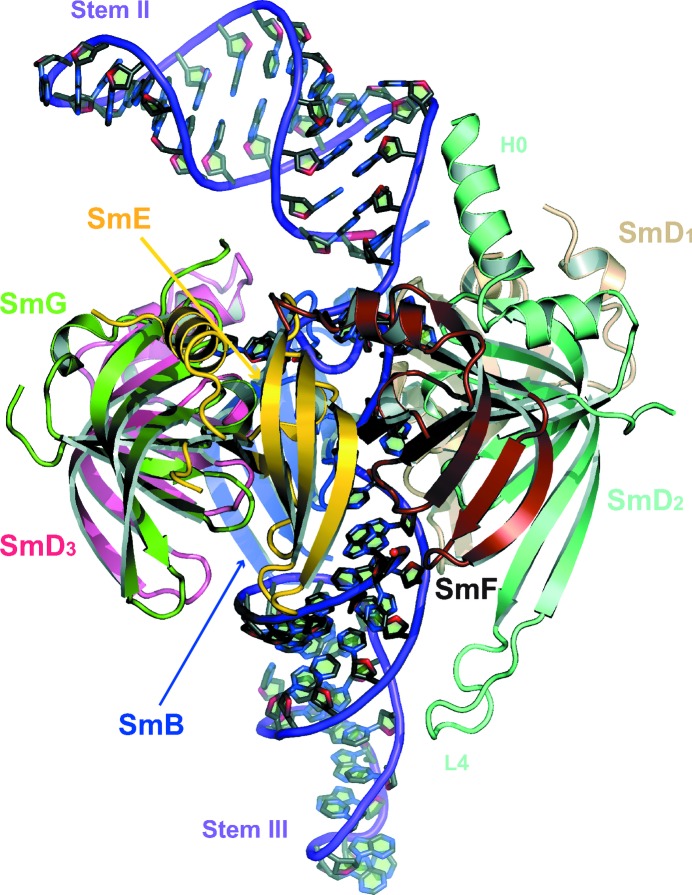
Cartoon of the U4 core domain. The re-refined model confirmed the architecture described by Leung *et al.* (2011 ▸). The seven Sm proteins form a ring by β4–β5 hydrogen bonding across every subunit interface. The protein order viewed from the flat face (top) of the ring is (clockwise) SmF–SmE–SmG–SmD_3_–SmB–SmD_1_–SmD_2_ (Kambach *et al.*, 1999 ▸). The Sm-site nonanucleotides of U4 snRNA are bound inside the central hole flanked by Stem II on the flat face and Stem III on the tapered face. The N-terminus of helix H0 in SmD_2_ is in contact with the bend in Stem II. The lysine-rich L4 loop of SmD_2_ makes charge interactions with the phosphate backbone of Stem III.

**Figure 4:**
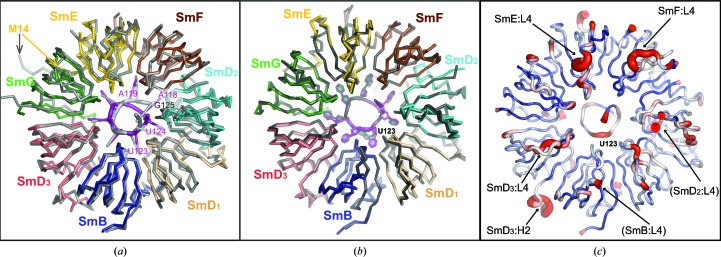
Structural comparisons in the core region between the re-refined U4 core domain and U4prev (*a*) and between it and the minimal U1 snRNP (*b*, *c*). The backbone (C^α^ and P atoms) superposition is viewed from the tapered face of the ring. The re-refined U4 structure is shown in (*a*) and (*b*) in the molecular colour code of Fig. 3 ▸, while U4prev in (*a*) and U1 in (*b*) are coloured grey. In (*a*), the pocket in SmF is correctly occupied by A118 of U4 snRNA (magenta ladder) but is incorrectly occupied by G125 in U4prev (grey ladder). The register of the remaining Sm-site nucleotides relative to the Sm proteins was correct. The sequence-register error at the N-terminus of SmE in U4prev is now corrected. (*b*) shows the close agreement between U4 and U1 snRNP in the core region, except at the G→U substitution (U123 in U4 snRNA). (*c*) illustrates the U4–U1 backbone differences in the core region displayed as a colour ramp of pseudo-*B* factors along the U4 backbone trace. The colour ranges from blue for differences less than 0.2 Å, passing through white, to red for differences above 1.0 Å. The backbone differences are localized to the N- and C-termini of the proteins and at the U/G substitution in the Sm site. Loops L4 of SmB and SmD_2_, which are divergent among NCS copies of the U4 structure, have been omitted from all of the comparisons.

Fig. 3 ▸ shows a cartoon of the re-refined U4 core-domain structure (PDB entry 4wzj). This is the untwinned model, which, in combination with the description of twinning, accounts for the observed diffraction (Table 1 ▸; Fig. 1 ▸). A least-squares superposition of 4wzj on U4prev (Leung *et al.*, 2011 ▸) shows their secondary structures to be matched with an r.m.s.d. of 1.07 Å between the aligned C^α^ and P atoms before and after re-refinement (Fig. 4 ▸ *a*). Compared with U4prev (Leung *et al.*, 2011 ▸), both L1 and L2 of SmE are now two residues longer, and A118 has replaced G125 as the nucleotide bound in SmF. In the refinement process summarized below, large *R*-value decreases occurred after molecular replacement using search models containing the corrected SmE (§§2.2–2.3) and after rebuilding the RNA stems from the corrected ends of the Sm site (§§2.4–2.5). The final *R*_work_ and *R*_free_ values of 17.7 and 22.4%, respectively, at 66.1–3.6 Å resolution represent a significant improvement from the values of 27.7 and 32.1%, respectively, for U4prev. Concomitantly, the *MolProbity* score (Chen *et al.*, 2010 ▸) relative to structures at similar resolution has risen from the 90th percentile for U4prev to the 100th percentile. These statistics indicate an improvement in model accuracy.

The re-refinement began with molecular replacement (§[Sec sec2.2]2.2) from the core region of the minimal U1 snRNP (Kondo *et al.*, 2015 ▸; PDB entry 4pjo) consisting of the seven Sm proteins each truncated to the Sm fold and the U1 snRNA truncated to its Sm-site nonanucleotide. The 4pjo model itself had been obtained by molecular replacement from the protein part of U4prev; however, the model was completed after density modification and refined to an *R*_work_ and *R*_free_ of 20.7 and 25.5% in the resolution range 70.0–3.3 Å, so that the bias towards U4prev was minimal. As an alternative to the 4pjo-derived search model, we also constructed a hybrid model by replacing the SmF–SmE–SmG sector of U4prev by its counterpart from 4pjo. The two search models differ principally in the sequence and conformation of the RNA nonamer in the central hole. Following parallel molecular replacement and brief refinement using *REFMAC*5, two RNA-omit maps were calculated in parallel and used to rebuild the bound nonamer in U4 snRNA (§[Sec sec2.4]2.4). The flanking RNA stems were added (§[Sec sec2.5]2.5) by joining one example of Stem II and Stem III that had been in intermolecular contact in U4prev onto the nona­nucleotide in all of the NCS copies. These stems diverged into their final conformations over many cycles of refinement in *REFMAC*5 under jelly-body restraints and external restraints (Murshudov *et al.*, 2011 ▸; Nicholls *et al.*, 2012 ▸). Therefore, the re-refined U4 core-domain structure has assimilated information from the previous model U4prev as well as the homologous structure of the minimal U1 snRNP. The statistics in Table 1 ▸ show that a good agreement with the data and good stereochemistry for both proteins and RNA have been achieved.

**Figure 5:**
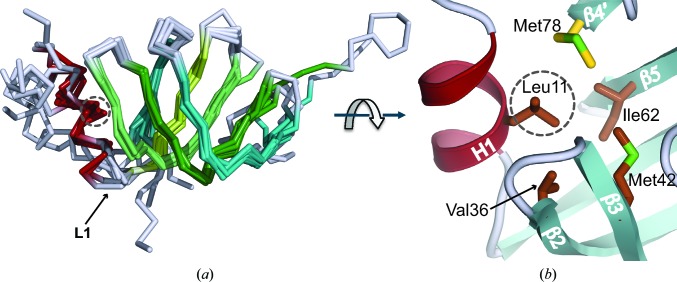
Structural conservation over the Sm fold. (*a*) Superposition of the C^α^ trace of all seven Sm proteins, with helix H1 coloured dark red and the β-strands in shades of cyan and green. A dashed circle indicates the C^α^ position of the conserved hydrophobic residue in H1. (*b*) The side chain-to-side chain contacts between the conserved residue in H1 and other conserved residues within the hydrophobic belt. This example shows contacts between Leu11 (in a dashed circle) in H1 of SmF and other residues of SmF (brown) and SmE (yellow).

Throughout the re-refinement, the 12 NCS copies of the U4 core domain in the asymmetric unit were subjected to local NCS restraints, which were imposed on the assembly of seven Sm proteins and one U4 snRNA fragment collectively. The final local NCS difference between protein copies was 0.016 Å on average (0.03–0.127 Å between different pairs); this difference between the RNA copies was 0.082 Å on average (0.035–0.121 Å between different pairs). These figures show a well defined consensus for the U4 core-domain structure, even when there are local differences among copies of the protein chains which are caused by packing contacts between neighbouring complexes, such as contacts between the β4 strands of SmD_2_ and SmF belonging to different complexes or between loop L4 of SmD_2_ and Stem III of RNA. The larger difference among copies of the snRNA reflects the variable curvature of the flanking stems, particularly Stem II, which allow the engineered tetraloop and tetraloop-receptor modules to make the contacts responsible for crystal formation (Leung *et al.*, 2010 ▸). Among these variations, the conformation of U4 Stem II in one NCS copy from PDB entry 4wzj closely modelled the density of the U4 3′ stem-loop at the interface with Brr2 (Nguyen *et al.*, 2013 ▸) from the U5 snRNP in a single-particle electron cryo-microscopy reconstruction of the yeast U4/U6·U5 tri-snRNP (Fig. 5 ▸ *b* in Nguyen *et al.*, 2015 ▸).

### 3.2. Consensus with the minimal U1 snRNP   

The re-refined structure of the U4 core domain is highly similar to the minimal U1 snRNP (Kondo *et al.*, 2015 ▸; PDB entry 4pjo) within the Sm fold of the seven Sm proteins and the Sm site of their snRNA. Fig. 4 ▸(*b*) shows the close superposition of their backbone (C^α^ and P) traces, which have an r.m.s.d. of 0.55 Å after averaging over the NCS copies. In Fig. 4 ▸(*c*), the backbone differences are expressed as pseudo-*B* factors along the sequence. The similarity is evident over the continuous β-sheet around the ring and in the central hole where the Sm site is bound, while differences above 1 Å are localized to the L4 loops of SmD_3_, SmE and SmF and at the U-to-G substitution in the Sm site. These exceptions from similarity between the core of the two structures are invariably owing to contacts with elements outside the core region. For example, the large conformational difference in L4 of SmD_3_ is owing to contacts of this loop, which are with the 3′ strand of RNA Stem III in the U4 core domain (§[Sec sec3.5]3.5) but with residues 21–24 of the N-terminal peptide of U1-70K in the minimal U1 snRNP (Kondo *et al.*, 2015 ▸). L4 of SmE and SmF interact with the 3′-terminus of U4 Stem III (§3.5), but are exposed to solvent in the minimal U1 snRNP (Kondo *et al.*, 2015 ▸). The backbone difference at U123 of the Sm site accompanies the relocation of the equivalent G of U1 snRNA (§[Sec sec3.4.2]3.4.2). Since localized systematic differences exist after refinement, it is unlikely that the overall similarities resulted from model bias. In the next sections, we describe the conserved architecture of the Sm fold and the conserved mode of recognition for the Sm-site nucleotides by the seven Sm proteins collectively, which underlie the similarity of the core structures.

### 3.3. Structural conservation over the Sm fold   

Superposition of the C^α^ traces of different Sm proteins in the U4 core domain (Fig. 5 ▸ *a*) demonstrates a striking structural conservation over the entire Sm fold, including the N-terminal helix H1. In SmD_2_ and SmE, where loop L1 is longer by two residues than in the other five Sm proteins, the longer L1 loops are also aligned with the superposition. As previously established, when neighbouring subunits are hydrogen-bonded between their β4 and β5 strands to form the ring, the ring is stabilized by a continuous hydrophobic belt of conserved inward-pointing residues. An additional stabilization is observed in the re-refined U4 core domain and the minimal U1 snRNP owing to H1 making van der Waals contacts on the flat face with the two hydrogen-bonded neighbours. In the heterodimers (Kambach *et al.*, 1999 ▸) only H1 from one of the monomers can make such van der Waals contacts and hence the disposition of H1 relative to the β-sheet is variable when the Sm proteins are not part of a complete ring (Collins *et al.*, 2001 ▸). In the closed ring of the re-refined U4 core domain H1 shows a more consistent orientation relative to the β-sheets and inserts a conserved hydrophobic residue into the hydrophobic belt (Figs. 5 ▸ *b* and 6 ▸ *a*). For example, in SmF Leu11 inserted from H1 contacts Val36, Met42 and Leu62 of SmF and Met78 of SmE. Val36 and Met42 flank L3 of SmF, and Leu62 is in β5 of SmF opposite Met78 in β4 of SmE (Fig. 5 ▸ *b*). Thus, H1 helps to stabilize the RNA-binding L3 loop (§3.4.2) as well as the intermolecular β-sheet of the ring.

### 3.4. Structural basis of Sm-site recognition   

#### 3.4.1. Register of the Sm site relative to the Sm proteins   

The segment of U4 snRNA buried inside the central hole of the core domain precisely matches the nonameric U4 Sm-site sequence 118-AAUUUUUGA-126 sufficient to assemble the seven Sm proteins *in vitro* into a ring with the same diameter and chemical sensitivity as the core domain with the full-length snRNA (Raker *et al.*, 1999 ▸). Likewise, the segment buried in the hole of the minimal U1 snRNP (Kondo *et al.*, 2015 ▸) is the equivalent U1 Sm-site sequence 125-AAUUUGUGG-133. In both the U4 and U1 Sm-site nonamers, the first seven nucleotides are each held in a single-protein pocket formed by the L3 and L5 residues from SmF–SmE–SmG–SmD_3_–SmB–SmD_1_–SmD_2_, respectively (Fig. 7 ▸), whereas the last two nucleotides are bound less intimately, being fitted between SmD_1_ and SmD_2_ and between SmE and SmF, respectively. Their distinct binding modes explain why the U4 Sm-site sequence minus the last two nucleotides assembled the seven Sm proteins slightly more efficiently (Raker *et al.*, 1999 ▸). The first seven nucleotides will be termed the Sm-site heptad.

Assembly with U1, U2, U4 or U5 snRNA was shown *in vitro* to proceed *via* an intermediate containing SmD_1_, SmD_2_, SmE, SmF and SmG, which can bind snRNA to form a subcore (Raker *et al.*, 1996 ▸), and a crescent-shaped pentamer of SmD_1_–SmD_2_–SmF–SmE–SmG has been seen in crystal structures of assembly intermediates (Zhang *et al.*, 2011 ▸; Grimm *et al.*, 2013 ▸). The nucleotide register on the Sm proteins in the U4 core domain suggests that the subcore binds 118-AAU-120 in SmF–SmE–SmG and 123-UU-124 in SmD_1_–SmD_2_, thereby orientating 121-UU-122 appropriately for binding by SmD_3_-SmB, leading to ring completion.

#### 3.4.2. Binding pockets for the Sm-site heptad   

**Figure 6:**
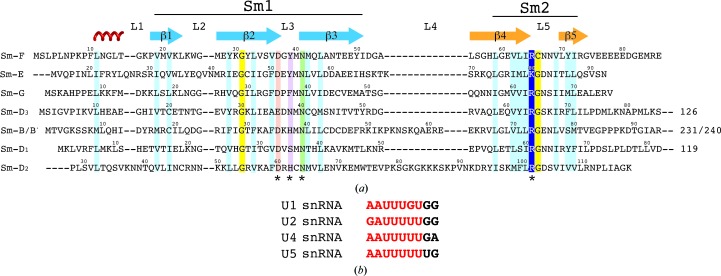
Structure-based sequence alignments. (*a*) The Sm protein sequences aligned by the superposition in Fig. 5 ▸(*a*). Conserved hydrophobic residues are highlighted in cyan and highly conserved glycines in yellow. Residues important for nucleotide binding are indicated with an asterisk and highlighted in red (Asp or Glu), magenta (mostly aromatic), green (invariant Asn) and blue (Arg or Lys). (*b*) Sm-site nonanucleotides of human U1, U2, U4 and U5 snRNA. The heptad bound one-to-one by the seven Sm proteins is shown in red.

Fig. 8 ▸ illustrates the seven binding pockets for the nucleotides of the U4 Sm-site heptad 118-AAUUUUU-124. The binding pockets are lined by the side chains of key residues at equivalent positions in the L3 and L5 loops of the Sm fold. L3 has a consensus sequence of acidic–hydrophilic–aromatic–Met–Asn, with Asn being invariant, and L5 has Arg–Gly–acidic/Asn (Fig. 6 ▸ *a*). The key residues providing side chain-to-base contacts are the Asn and aromatic residues in L3 and Arg in L5. The Asn side chain in L3 forms hydrogen bonds to the base in all seven pockets. Orientation of the Asn side chain in L3 is maintained by the conserved hydrogen bonds that buttress the L3 and L5 loops (Kambach *et al.*, 1999 ▸; Törö *et al.*, 2001 ▸; Zhang *et al.*, 2011 ▸; Grimm *et al.*, 2013 ▸). Upon RNA binding the Asn and aromatic side chains in L3 both remain in the same rotamer as in the apo form. Hence, the consensus structure in L3 and L5 determines how the bases are bound.

The binding of the U-tract (120-UUUUU-124) shows analogy with the binding of penta-uridylate by *Archaeoglobus fulgidus* Lsm-1 (Törö *et al.*, 2001 ▸) to varying degrees, while the structural motif for U recognition is conserved among archaeal Lsm proteins (Mura *et al.*, 2003 ▸). In *A. fulgidus* Lsm-1, the L3 and L5 loop sequences of which follow the same consensus patterns (Fig. 6 ▸ *a*) as the Sm proteins, the uridine base is sandwiched between the His and Arg side chains and hydrogen-bonded to the invariant Asn side chain (Törö *et al.*, 2001 ▸). Similarly, in the U4 Sm site the binding pockets for U120 in SmG, U122 in SmB and U124 in SmD_2_ provide U-specificity by sandwiching the uridine base between the aromatic and Arg side chains, and by hydrogen-bonding of the O^δ1^ and N^δ2^ atoms of the invariant Asn to N3 and O4, respectively, on the uridine base (Figs. 8 ▸ *c*, 8 ▸ *e* and 8 ▸ *g*). These contacts account for the UV cross-linking of the first and third U with the L3 residues of SmG and SmB, respectively (Urlaub *et al.*, 2001 ▸). In the binding pocket for U121 in SmD_3_, the planar amide group of Asn38 replaces the aromatic side chain, and together with the Arg64 side chain sandwiches the base, while the invariant Asn40 forms the two U-specific hydrogen bonds with the base (Fig. 8 ▸ *d*). In SmD_1_, Ser35 replacing the aromatic residue cannot provide stacking, so that the U123 base is only stacked with the Arg61 side chain and forms one hydrogen bond from N3 to O^δ2^ of Asn37 (Fig. 8 ▸ *f*). In all five pockets O2 of uridine is hydrogen-bonded to a peptide N atom in L5, which stabilizes the binding.

In the U1 Sm site G occupies the position equivalent to the fourth U (Fig. 6 ▸ *b*; Burge *et al.*, 2012 ▸). The larger guanine base is located outside the central hole by a rotation of the backbone and lies in the *syn* conformation in contact with Lys36 in L3 of SmB (Kondo *et al.*, 2015 ▸). His37 of SmB is stacked with the third U in both the U4 and U1 Sm sites, but Urlaub *et al.* (2001 ▸) found that UV irradiation cross-linked L3 residues of SmB to the third U only in the U4 Sm site. The lack of photo-cross-linking in the U1 Sm site is most likely because of shielding by the adjacent G130 base (Fig. 9 ▸ *a*). Other than at this position, the heptads of the U4 and U1 Sm sites are identical in sequence and superpose closely (Figs. 4 ▸ *b* and 9 ▸ *a*).

The binding pockets for the two adenines at the 5′-end of the Sm site are no larger than those for the U-tract, which is consistent with the conserved protein structure in L3 and L5. However, the phosphates of A118 and A119 are located farther from the side chain of the invariant Asn in L3 of SmF and SmE, respectively, than the phosphates in the U-tract are from the corresponding Asn side chains (Fig. 9 ▸ *b*), and this causes purines to be preferred for binding in the pockets in SmF and SmE. By contrast, the G in the U1 Sm site that is located outside the SmD_1_ pocket has a smaller phosphate-to-Asn distance than the fourth U of the U4 Sm site that it replaces (Fig. 9 ▸ *b*). Such comparisons point out the importance of the irregular backbone conformation of the Sm site in determining the base preference of the binding pockets. Each adenine base is stacked with a tyrosine in L3 and forms one hydrogen bond from N1 to N^δ2^ of the invariant Asn (see below), showing similarities with the U-tract. *In vitro*, a nona-uridylate can assemble the seven Sm proteins into a ring, but the product lacks thermal stability (Raker *et al.*, 1999 ▸), which is presumably owing to the absence of the base–Asn hydrogen bond in the first two pockets and confirms the influence of the backbone conformation in base selection.

**Figure 7:**
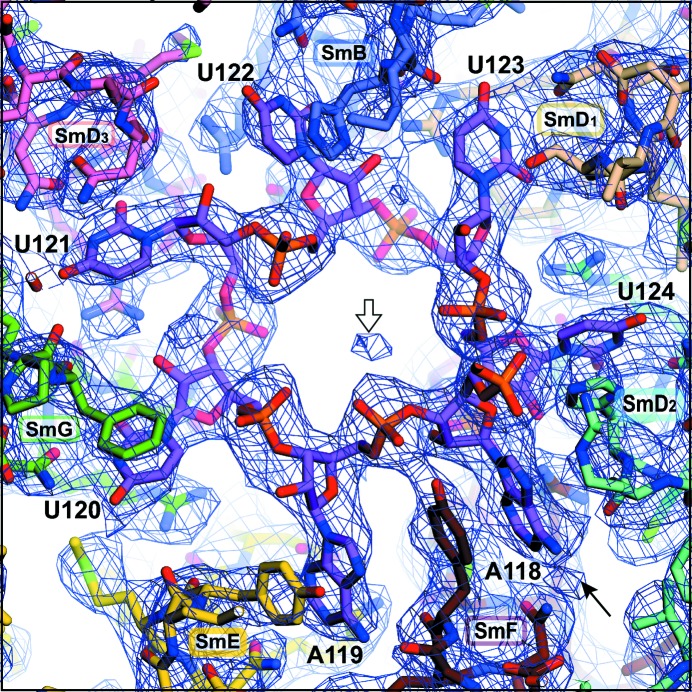
2*mF*_o_ − *DF*_c_ map of the Sm-site region superimposed on the re-refined structure. The electron-density map is averaged over the 12 NCS copies after sharpening in *REFMAC*5 (*B* = −16.38 Å^2^ for simple map sharpening; *B* = −61.47Å^2^, α = 0.0235 for regularized map sharpening) and contoured at 0.54 e Å^−3^. The superposition shows that each base of the U4 Sm-site heptad (118-AAUUUUU-124) is bound in one Sm protein. The white arrow in the central hole points to the density feature at the equivalent location to a hydrated Mg^2+^ ion in the U1 Sm site. The black arrow at the lower right points to the solvent-exposed N6 atom of A118.

**Figure 8:**
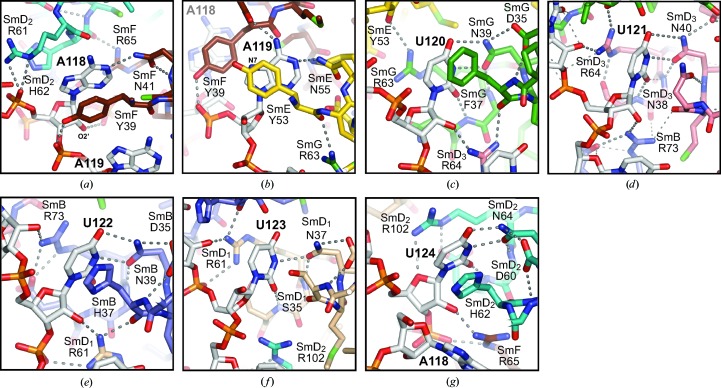
Interactions of the U4 Sm-site heptad with the Sm protein-binding pockets. (*a*) A118 bound in SmF: O2′ of A118 is hydrogen-bonded to the OP1 atom of G125 in the background. (*b*) A119 bound in SmE: N7 of A119, which is methylated by DMS, is located near the axis of the π-system of Tyr53 of SmE, whereas N7 of A118, which is not methylated by DMS, is distant from the axis of the π-system of Tyr39 of SmF. (*c*) U120 bound in SmG. (*d*) U121 bound in SmD_3_. (*e*) U122 bound in SmB. (*f*) U123 bound in SmD_1_. (*g*) U124 bound in SmD_2_. See the text for details of the contact residues.

In SmF, A118 is stacked with Tyr39 in L3 and stabilized by the hydrogen bond between O^η^ of Tyr39 and OP2 of A119; it is hydrogen-bonded from N1 to N^δ2^ of Asn41 (Fig. 8 ▸ *a*). Arg65 in L5 does not contact this base but forms hydrogen bonds to the backbone of U124 and G125 (not shown). N6 of A118 (Fig. 7 ▸, black arrow) is accessible to solvent for polar interactions, which allows the first A to be replaced by G in the Sm site of U2 snRNA (Fig. 6 ▸ *b*). A119, the second adenine, is stacked with Tyr53 in L3 of SmE, and is hydrogen-bonded from N1 to N^δ2^ of Asn55 in L3 of SmE and from N6 to the carbonyl O atom of Gly38 in L3 of SmF (Fig. 8 ▸ *b*). The latter hydrogen bond makes the second A irreplaceable by G (Raker *et al.*, 1999 ▸). Lys80 in L5 of SmE makes no base contacts but is hydrogen-bonded from N^ζ^ to the carbonyl O atom of Cys66 in L5 of SmF (not shown).

Hartmuth *et al.* (1999 ▸) observed that dimethyl sulfate (DMS) methylated the N7 atom of the second A upon assembly of the core domain or its five-Sm-protein intermediate. They hypothesized, in the absence of structural information, that protein binding distorted the π-electron delocalization over the double ring to create an unusual nucleophilic centre at N7. However, the two hydrogen bonds to the A119 base cannot significantly distort its electronic delocalization, and neither is such distortion required for the nucleophilic activity. Instead, N7 methylation will be facilitated by stabilization of its transition state(s), during which positive charge develops on the N7 atom. Our structure and that of the minimal U1 snRNP (Kondo *et al.*, 2015 ▸) suggest that the geometry of stacking between A119 (A126 in U1 snRNA) and Tyr53 of SmE (Fig. 8 ▸ *b*) allowed stabilization of the incipient positive charge on N7 through cation–π interaction with the π-system of the tyrosine. Interestingly, in native U1 snRNP an additional adenine, A135, at position +11 from the start of the Sm site is also N7-methylated by DMS (Hartmuth *et al.*, 1999 ▸), and the crystal structure of the minimal U1 snRNP shows A135 adjacent to Tyr38 of the U1-70K protein (Fig. 3*c* in Kondo *et al.*, 2015 ▸) with N7 near the axis of the π-system of tyrosine.

#### 3.4.3. Binding of the last two nucleotides   

The base plane of G125 fits between Lys20 in L2 of SmD_1_ and Arg47 at the equivalent position in L2 of SmD_2_ (Figs. 6 ▸ *a* and 10 ▸ *a*). The two contacting residues make multiple hydrogen bonds, whereas the base of G125 is only hydrogen-bonded from O6 to Arg66 in L5 of SmD_1_ (Fig. 10 ▸ *a*), which is consistent with this pocket not being specific for G. In U5 snRNA from the majority of species, including humans, U is found at this position (Fig. 6 ▸ *b*), which could form a hydrogen bond from O4 to Arg66. The base of A126 is held in edge-to-face fashion between the edges of Trp25 in L2 of SmF and Tyr36 in L2 of SmE; its sugar edge points to the first base pair of Stem III (Fig. 10 ▸ *b*). This base forms no hydrogen bonds to the proteins, and in human U1, U2 and U5 snRNA this pocket contains G instead of A. Superposition of the U4 core domain on the minimal U1 snRNP shows that the bases of A126 in U4 snRNA and the equivalent G133 in U1 snRNA overlap in space, although one is in the *anti* and the other is in the *syn* conformation.

#### 3.4.4. Summary of Sm-site recognition   

**Figure 9:**
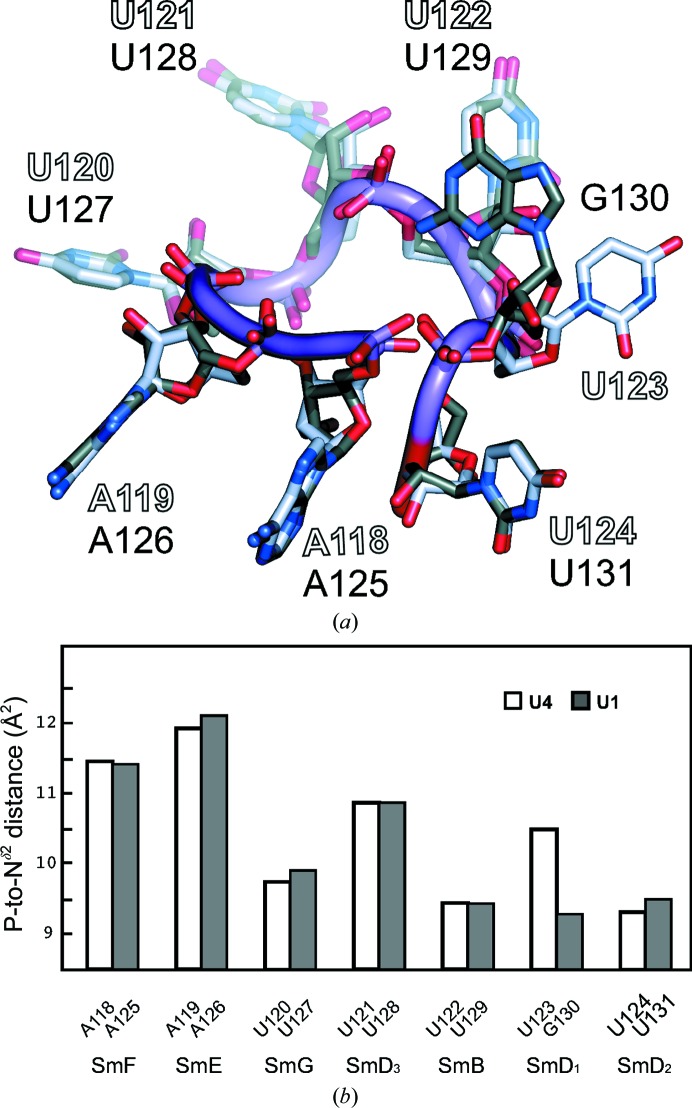
Comparison of the U4 and U1 Sm-site heptads. (*a*) The U4 and U1 Sm-site heptads are superimposed and viewed from the flat face at a glancing angle. The only notable difference occurs at the sixth nucleotide, where the U123 base of U4 snRNA is bound in the pocket of SmD_1_, while the G130 base of U1 snRNA lies outside the central hole. The G130 base partially covers the base of U129, which is stacked on the face near G130 with His37 in L3 of SmB. Thus, shielding of UV irradiation by G130 is likely to have prevented the cross-linking of U1 snRNA to L3 of SmB (Urlaub *et al.*, 2001 ▸). (*b*) Distances from the P atom of the Sm-site nucleotide to the N^δ2^ atom of the invariant Asn in its binding pocket. The P-to-N^δ2^ distance measures the RNA backbone position relative to the depth of the pocket, where the base is hydrogen-bonded to N^δ2^. The distances are NCS-averaged with standard deviations of 0.04–0.10 Å at each position. The distances are closely similar between the U4 and U1 Sm sites in all except the sixth pocket and they are longer for A than for U. In the sixth pocket, G130 of U1 snRNA, which shows a shorter P-to-N^δ2^ distance than U123 of U4 snRNA, is excluded from the SmD_1_ pocket.

**Figure 10:**
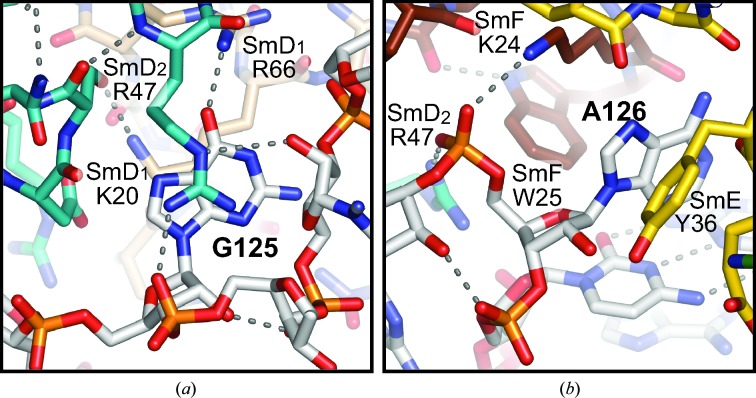
Binding of the 3′-most nucleotides of the U4 Sm site. (*a*) G125 bound between SmD_1_ and SmD_2_. (*b*) A126 bound between SmE and SmF.

Our observations of the binding pockets for the U4 Sm-site nonamer accounted for the relative stringencies in their base preference, and therefore we have reached a coherent account of the structural basis of Sm-site recognition. The binding pockets for the first seven nucleotides (the heptad) are inter-linked by hydrogen bonds between protein residues lining adjacent pockets, between these residues and the ribose phosphate backbone and between the backbone atoms of successive nucleotides (Fig. 8 ▸). Such links stress the importance of the backbone conformation, as previously probed by Raker *et al.* (1999 ▸), which is ultimately consistent with the recognition of the Sm site by the Sm proteins collectively. The superposition between the U4 and U1 Sm-site heptads (Fig. 9 ▸ *a*) showed that, except at one position of sequence difference, their backbones adopt the same irregular conformation and their nucleotide bases have identically varied orientations. The variation of base orientation is accompanied by small variations in the orientation of the binding loops in different Sm proteins. In the central hole, the NCS-averaged 2*mF*_o_ − *DF*_c_ map of the Sm-site region (Fig. 7 ▸) shows a density feature (white arrow) at the equivalent location to a hydrated Mg^+2^ ion that stabilizes adjacent phosphate groups of the U1 Sm site (Kondo *et al.*, 2015 ▸). However, bound ions have not been modelled for the U4 Sm site because of the limited resolution.

### 3.5. Protein interactions with the U4 snRNA stems   

Stem II of U4 snRNA nearest the Sm site consists of three G–C pairs (residues 85–87 and 115–117) and the bulged U114; the stem then turns away from SmD_1_ and towards SmG (Fig. 3 ▸). Contacts with Sm proteins occur around the bend. In the 5′ strand, C86 and C87 contact Lys36 of SmB and Lys3 of SmG, respectively. In the 3′ strand, C115 and C116 contact Leu16 and U114 contacts Pro13, which are from the N-terminus of helix H0 of SmD_2_. H0 of SmD_2_ is linked to H1 by a tight turn and its orientation relative to H1 is similar in the absence and presence of RNA (Grimm *et al.*, 2013 ▸; Kondo *et al.*, 2015 ▸), but its N-terminal region is more ordered when in contact with RNA (Fig. 3 ▸). In U1 snRNP, the N-terminus of H0 points into the minor groove of helix H, buttressing the U1 snRNA (Pomeranz Krummel *et al.*, 2009 ▸). In most copies of the U4 core domain, because of the variable curvature of Stem II, H0 does not contact the snRNA and is disordered towards its N-terminus. While the length of H0 is variable, the orientation of H0 relative to H1 of SmD_2_ and to the Sm ring is consistent among NCS copies in PDB entry 4wzj and with the minimal U1 snRNP structure. The conformation of H0 of SmD_2_ reported in Leung *et al.* (2011 ▸) was a misinterpretation.

Stem III of U4 snRNA nearest the Sm site consists of a helix of seven Watson–Crick base pairs (nucleotides 127–133 and 138–144), after which the tetra-loop receptor for crystallization purposes is inserted (Leung *et al.*, 2010 ▸). This helix of the natural sequence accounts for most of the extensive protein contacts with Stem III. Counting away from the ring, its 5′ strand contacts L2 of SmD_2_, SmF and SmD_1_ before L4 of SmD_2_; its 3′ strand contacts L2 of SmE and SmF before L4 of SmE and SmB. Contact residues in L4 include five Lys residues (79, 84, 85, 86 and 88) from SmD_2_, Lys67 of SmE and Arg49 of SmB; these basic side chains interact with phosphate groups of Stem III from the same copy or an adjacent copy. In the absence of RNA, L4 of SmD_2_ is disordered (Zhang *et al.*, 2011 ▸; Grimm *et al.*, 2013 ▸), while L4 of SmB is relatively ordered through crystal contacts (Kambach *et al.*, 1999 ▸). In the U4 core domain, L4 of SmD_2_ becomes ordered in the conformations favourable to electrostatic interactions with the RNA backbone, whereas L4 of SmB in most copies becomes disordered after Arg49 from a lack of RNA contacts.

The 3′-helix in U4 (Stem III) and U5 snRNA flanks the Sm site directly, without an intervening single-stranded stalk such as exists between the U1 Sm site and its 3′-helix (Burge *et al.*, 2012 ▸). Leung *et al.* (2011 ▸) deduced that the N-terminal peptide of U1-70K that wraps around the tapered face of the U1 core domain bypassing the single-stranded stalk (Pomeranz Krummel *et al.*, 2009 ▸; Kondo *et al.*, 2015 ▸) could not follow an equivalent path on the U4 core domain because of obstruction by Stem III. The re-refined model predicts obstruction by nine nucleotides in Stem III. Thus, the architectural difference of the 3′-helix underlies the selective binding of the U1-70K protein to the U1 snRNP core domain and its exclusion from the noncognate U4 and U5 snRNP. This interpretation is supported by the failure of a 90-residue N-terminal peptide of U1-70K to bind to the U5 core domain (Nelissen *et al.*, 1994 ▸). Overall, the different snRNAs selectively stabilize different protein conformations and permit the binding of accessory proteins that distinguish the various snRNPs.

## 4. Discussion   

### 4.1. The two-stage approach to structure determination extended to three stages   

**Figure 11:**
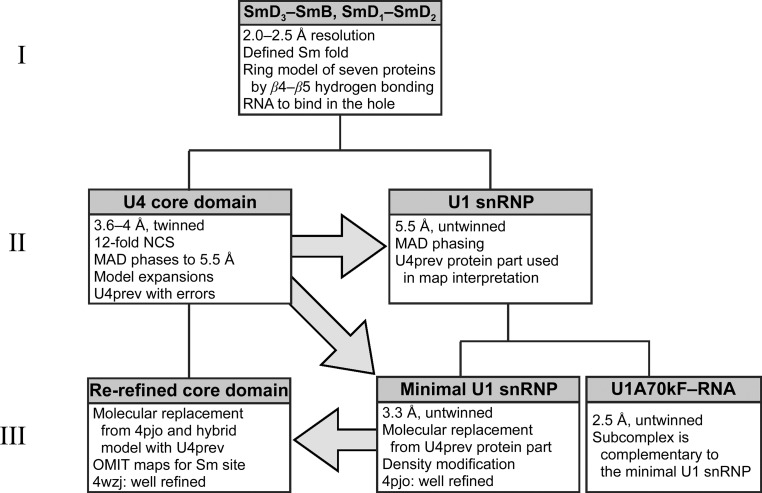
Hierarchical diagram of structure determination for the U4 core domain. In stage I, the structures of two Sm-protein heterodimers were solved, which defined the Sm fold for the protein family and led to a ring model for the organization of seven Sm proteins in the snRNP core domain (Kambach *et al.*, 1999 ▸). In stage II, structures of the U4 core domain (Leung *et al.*, 2011 ▸) and the U1 snRNP (Pomeranz Krummel *et al.*, 2009 ▸) were solved, with the latter containing the core domain plus two accessory proteins. The protein part of the U4 structure helped to interpret the U1 map (Oubridge *et al.*, 2009 ▸). However, twinning of the U4 crystals prevented the determination of an accurate structure, and the U1 snRNP structure at limited resolution could not resolve the mechanism of 5′ splice site recognition. In stage III, two subcomplexes designed from the 5.5 Å resolution structure of U1 snRNP crystallized well. Of these, the minimal U1 snRNP is closely homologous to the U4 core domain; its structure was determined by molecular replacement from the protein part of the previous U4 structure (U4prev) and the model was completed after density modification (Kondo *et al.*, 2015 ▸). Using the core of minimal U1 snRNP, and a hybrid model from this and U4prev as alternative models for molecular replacement, the U4 core-domain structure was re-refined. An OMIT map from the hybrid model proved that the Sm-site nucleotides are bound in the same manner in the two core domains except at the one position where their sequences differ.

Structure determination of large complexes often succeeds in two stages: the structures of individual components or subcomplexes are solved to high resolution in the first stage and are then used in the second stage to solve the large complex by molecular replacement or to help to interpret an experimentally phased electron-density map at lower resolution. We first solved the structures of the SmD_3_–SmB and SmD_1_–SmD_2_ heterodimers to 2.0 and 2.5 Å resolution, respectively, from which we identified the conserved Sm fold and proposed a ring model for the organization of the seven Sm proteins in the core of snRNPs (Kambach *et al.*, 1999 ▸). In order to discover the RNA-binding interactions in the snRNP core, we proceeded to crystallize the U4 core domain (Leung *et al.*, 2010 ▸). Unfortunately, well diffracting and untwinned crystals of the U4 core domain, which would have ensured the accuracy of the structure determined at 3.6 Å resolution (Leung *et al.*, 2011 ▸), were not obtained. However, extending the investigation to a third stage, in which our preliminary interpretations were exploited to generate better diffracting crystals of subcomplexes of the homologous U1 snRNP, eventually allowed us to improve the U4 core-domain structure. Our tortuous route to a well refined structure of the core domain is sketched out in a hierarchical diagram of structural determinations (Fig. 11 ▸).

### 4.2. Path to the previous U4 core-domain model   

Exhaustive efforts have been made to obtain useful diffraction data from the U4 core domain. U4 snRNA was chosen because its Sm site is immediately flanked by two RNA stem loops without the potentially flexible, single-stranded RNA links found in U1, U2 and U5 snRNA. Tertiary interaction modules were engineered into the snRNA stems distal from the Sm site in order to promote crystal contacts between complexes without perturbing the essential interactions of Sm-site recognition. Ten crystal forms were characterized. The *P*3_1_ crystal form (Leung *et al.*, 2010 ▸) enabled the initial solution of the structure at 3.6 Å resolution (Leung *et al.*, 2011 ▸) that we have now re-refined. In these crystals, each copy of the core domain was found to have four unique crystal contacts, of which only the designed interface (between a GAAA tetra-loop capping Stem II and the tetra-loop receptor incorporated in Stem III) showed stereospecific interactions through multiple hydrogen bonds, thus confirming the importance of the engineered interaction modules in achieving crystallization.

At the medium to low resolution typically achieved by crystals of large complexes, experimentally determined phases are critical to obtaining interpretable density maps. Initial phases for the U4 core domain (described under methods in the online version of Leung *et al.*, 2011 ▸) were obtained at 5.5 Å resolution by the MAD method using crystals labelled with selenomethionine (SeMet) in the SmE, SmF and SmG proteins. The low-resolution derivative data sets showed apparent *P*6_1_22 symmetry because twinning was masked by translational NCS and anisotropy. In addition, the threefold translational NCS (pseudo-symmetry) caused intensity modulations in the (*h*0*l*) zone, making two out of every three rows of reflections systematically weak. The heavy-atom parameters were refined by selecting the strong rows, and therefore the phases calculated corresponded to a reduced cell of one third of the volume and containing one core-domain complex per asymmetric unit, which effectively approximated all of the noncrystallographic symmetries as crystallographic. The distribution of SeMet anomalous difference peaks in the experimentally phased map verified the protein order around the ring. A partial model containing the seven proteins and a fragment of the 3′-stem was built in the reduced cell based on the crystal structures of SmD_3_–SmB and SmD_1_–SmD_2_ and homology models of SmE, SmF and SmG (Leung, 2005 ▸). The partial model was expanded by molecular replacement into the true cell with three copies per asymmetric unit in *P*6_1_22. Molecular replacement was repeated for different derivative data sets, and a multi-crystal averaged map was calculated by *DMMULTI* (Cowtan, 1994 ▸) and used to complete the model of the RNA. Subsequent analysis of the native intensities identified tetartohedral twinning and the space group was reclassified as *P*3_1_. The model reported at 3.6 Å resolution by Leung *et al.* (2011 ▸) was obtained after twin refinement in *REFMAC*5 under 12-fold NCS restraints against the native data. This model (*i.e.* U4prev) obtained by initial experimental phasing and stepwise relaxation of the symmetry approximations displayed the correct architecture of the core domain but contained model errors from the early maps.

### 4.3. Improving model accuracy *via* homologous structures   

The protein part of U4prev nonetheless helped model building into a 5.5 Å resolution map of the U1 snRNP obtained by experimental phasing (Pomeranz Krummel *et al.*, 2009 ▸; Oubridge *et al.*, 2009 ▸). These U1 snRNP crystals contained the U1 core domain made of the same seven Sm proteins and a truncated U1 snRNA molecule with two U1-specific proteins: U1-70K and U1-C. The 5.5 Å resolution structure explained why the U1-70K and Sm proteins are required for the association of U1-C with U1 snRNP. However, the resolution was insufficient to reveal how U1 snRNP recognizes the start of the intron in pre-mRNA.

Better diffracting crystals were obtained from two sub­particles of the U1 snRNP judiciously designed after studying interactions in the 5.5 Å resolution structure. They led to two structures at 2.5 and 3.3 Å resolution that together illustrate the network of interactions between all of the components of U1 snRNP (Kondo *et al.*, 2015 ▸). The structure at 3.3 Å resolution is that of the minimal U1 snRNP. It was solved by molecular replacement from the protein part of U4prev. The model was completed after density modification, and in the process the formerly homology-modelled subunits SmE, SmF and SmG were updated by comparison with the 1.9 Å resolution coordinates of PDB entry 4f7u (Grimm *et al.*, 2013 ▸). The refined structure of minimal U1 snRNP (PDB entry 4pjo) revealed the mechanism for 5′ splice site recognition and contains in its core region the closest structural homologue to the U4 core domain.

Using the common core from the minimal U1 snRNP, two alternative molecular-replacement probes were constructed to launch our re-refinement of the U4 core-domain structure. The resulting OMIT maps directed us to objectively correct previous model errors. Refinement was completed after rebuilding the U4 snRNA stems flanking the Sm-binding site, which differ from the U1 snRNA in these regions and also contain the contact modules engineered for crystallization. The validity of the re-refined model is supported by the improved statistics, consensus with the U1 snRNP structure and the ability to clarify some hitherto unexplained biochemical observations. Comparison between the two structures shows that the mechanism for specific recognition of the Sm site is conserved among U1, U2, U4 and U5 snRNPs, while conformational variations exist at the periphery of the core domain to allow interaction with the snRNP-specific proteins and snRNA. These two aspects of the 4wzj coordinates have made them useful for modelling other snRNPs from other species in electron cryomicroscopy maps of larger complexes from the splicing machinery, such as the *Saccharomyces cerevisiae* U4/U6·U5 tri-snRNP at 5.9 Å resolution (Nguyen *et al.*, 2015 ▸) and the *Schizosaccharomyces pombe* intron lariat spliceosome (ILS) at 3.6 Å resolution (Yan *et al.*, 2015 ▸).

In conclusion, using better homology models and new refinement tools, we have obtained a more accurate model of the U4 snRNP core-domain structure that gives a coherent account of the recognition of the Sm site by the seven Sm proteins collectively.

## Supplementary Material

Supplementary Figure 1.. DOI: 10.1107/S2059798315022111/kw5130sup1.pdf

## Figures and Tables

**Table 1:** Data-collection, refinement and model statistics Values in parentheses are for the outer shell.

PDB entry	4wzj
Data collection	
Wavelength (Å)	0.9795
Space group	*P*3_1_
Unit-cell parameters (Å, °)	*a* = *b* = 248.0, *c* = 251.9, α = β = 90.00, γ = 120.00
Resolution (Å)	68.20–3.47 (3.66–3.47)
*R*_merge_ (%)	20.9 (72.8)
Unique reflections	220236
Mean *I*/σ(*I*)	3.3 (1.1)
CC_1/2_	0.991 (0.234†)
Completeness (%)	98.6 (97.8)
Average multiplicity	2.0 (1.9)
Refinement‡	
Resolution (Å)	66.15–3.60 (3.69–3.60)
*R*_work_/*R*_free_	0.177/0.224 (0.260/0.270)
No. of reflections (*R*_work_/*R*_free_)‡	158528/8251 (3086/152)
Completeness‡ (%)	83.1 (21.8)
Twin operators and estimated twin fractions	
*h*, *k*, *l*	0.2177
−*k*, −*h*, −*l*	0.2825
*k*, *h*, −*l*	0.2833
−*h*, −*k*, *l*	0.2165
Model	
No. of atoms	
Total	71485
Protein	54046
RNA	17436
Water	3
R.m.s. deviations from ideal geometry§	
Bond lengths (Å)	0.0105
Bond angles (°)	1.399
*B* factors (Å^2^)	
Overall	124.4
Protein	118.4
RNA	162.8
Water	42.7
Ramachandran plot¶	
Favoured (%)	96.74
Outliers (%)	0.13
*MolProbity* †† score [percentile]	1.78 [100th]
All-atom clashscore [percentile]	11 [97th]
Good rotamers (%)	98.95
Good RNA backbone conformation (%)	98.16

† CC_1/2_ = 0.516 at 3.88–3.66 Å.

‡ Values after correction for diffraction aniso­tropy.

§ Using the *REFMAC*5 dictionary (Vagin *et al.*, 2004 ▸).

¶ Lovell *et al.* (2003 ▸).

†† Chen *et al.* (2010 ▸).
