# Supplementary material for: Re-refinement of the spliceosomal U4 snRNP core-domain structure
Source: Acta Crystallogr D Struct Biol. 2016 Jan 1;72(Pt 1):131–46. doi: 10.1107/S2059798315022111 (PMC4756616; doi:10.1107/S2059798315022111)
Supplement: Supplementary file 1 [file d-72-00131-sup1.pdf]

STRUCTURAL
BIOLOGY

Volume 72 (2016)

Supporting information for article:

Re-refinement of the spliceosomal U4 snRNP core-domain structure

Jade Li, Adelaine K. Leung, Yasushi Kondo, Chris Oubridge and Kiyoshi Nagai

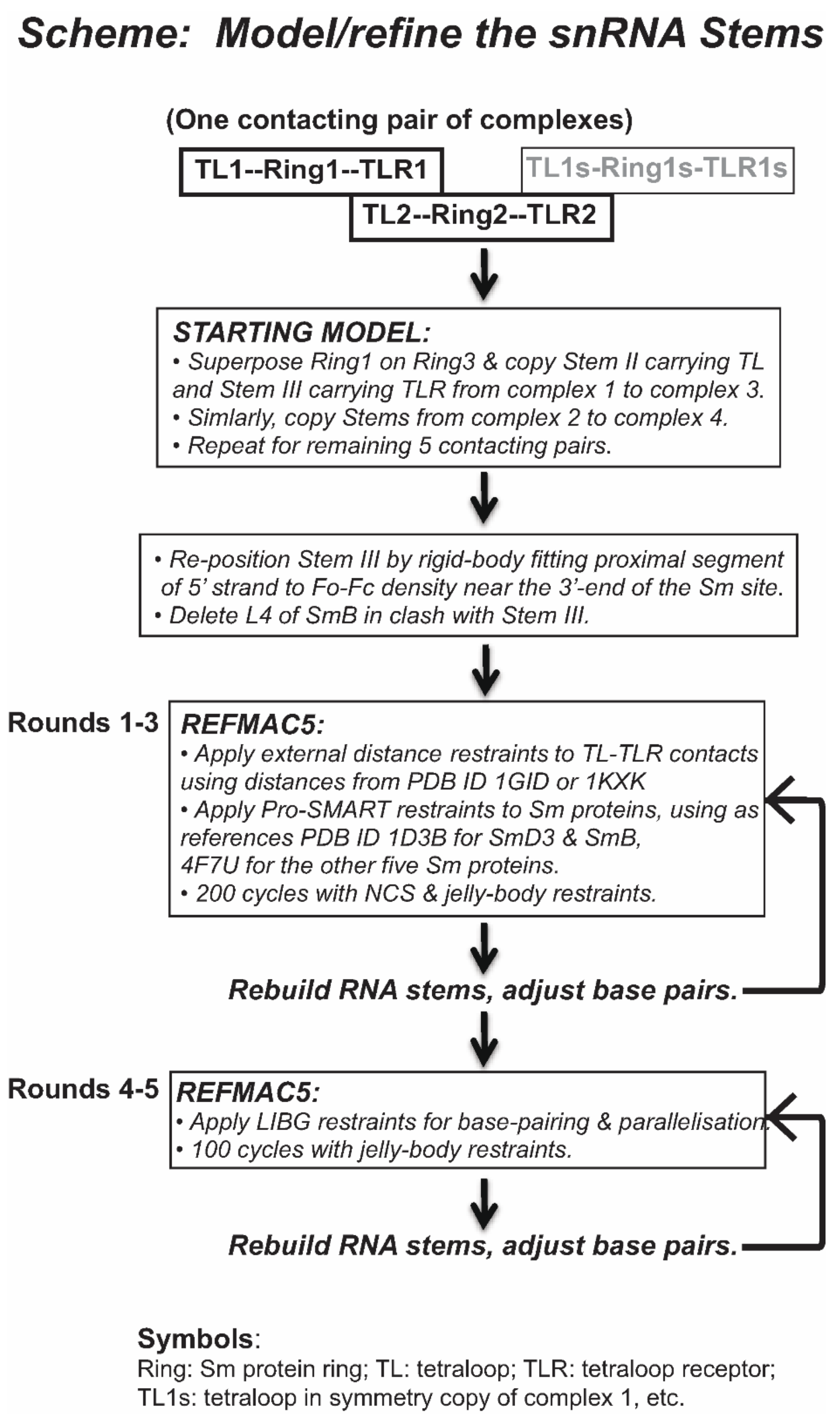

Figure S1 Steps used to re-model and refine the flanking stems in §2.5
